# Interactions of Highly Diluted *Arnica montana* Extract with Water Across Glass Interfaces †

**DOI:** 10.3390/ijms26031115

**Published:** 2025-01-27

**Authors:** Igor Jerman, Linda Ogrizek, Jonatan Pihir, Mateja Senica

**Affiliations:** BION Institute, Stegne 21, 1000 Ljubljana, Slovenia; linda.ogrizek@bion.si (L.O.); jonatan.pihir@bion.si (J.P.); mateja.senica@bion.si (M.S.)

**Keywords:** high dilution, HD interaction with water, physicochemical measurements, UV/VIS/NIR spectroscopy, HD signal transfer, *Arnica montana*

## Abstract

This study explores the physicochemical changes provoked by the physical transmission of highly diluted (HD) solutions of *Arnica montana* extract on three receiver solutions differing by their pH. Three dilutions (potencies), one modest (D6), one very high (C30), and another ultra-high (C200) extract of *A. montana*, were used as a source of HD signal transfer. The HD signal transfer was enhanced by an initial knocking at the start of the experiment and then allowed to interact with the receiver solution for 24 h of exposure. The results confirmed the detectability of the HD signal transfer in solutions with different pH, the general effect of this signal on pH increase, the differential effect of the signal depending on the initial dilution level (potency), and the effect on the decrease in both the electrical voltage in water (ORP) and the conductivity. The overall findings of the study offer valuable new insights and suggest innovative approaches for further research, particularly in detecting the HD signal in solutions with varying pH levels, focusing on interactions with hydroxide and hydronium ions.

## 1. Introduction

Numerous studies have focused on the unexpected performance of highly diluted (HD) solutions, characterized by concentrations of the original substance that are orders of magnitude below those anticipated to elicit chemical or biological effects. Rather than containing molecular constituents, these aqueous solutions may possess the molecular information of one or more compounds originally dissolved within them [1,2,3,4,5]. In our previous research, we worked with molecular information of antibodies to IFNγ (a-IFNγ) that were diluted in distilled water in different concentrations and then further processed [6,7]. This kind of stored molecular information will be named HD signal or HD molecular information. The interaction of this signal with the nearby water (solution) in the form of a signal transference from the original HD solution to the receiver solution will be termed the *signal transfer* [6,7]. Similar research approaches regarding HD signal processing have also been explored in other studies [8,9,10].

In two cited studies [6,7], we examined a potential detection of HD signals from initially diluted material, assumed to be present even at very high dilutions in distilled water. Three physicochemical methods (electrical conductivity, oxidation reduction potential (ORP), and pH) plus UV/VIS/NIR spectroscopy were employed in the studies. There, we investigated how high-dilution (HD) solutions of a-IFNγ alter the physicochemical properties of receiving water. The signal transfer occurred mechanically through glass and, notably, was also detected through air over two distinct distances. Encouraged by these findings, we extended our research to explore how the signal affects receiver liquids across three pH levels.

To avoid relying on a single source and to achieve greater generality while exploring the signal transfer properties of various receiver solutions, we opted to use a new source of molecular information—*Arnica montana*. Its tincture or extract is known for its beneficial biological effects [11,12,13], and it has been used for a number of health problems including physical injuries, pain relief, and treatment of inflammatory conditions [14,15]. Of particular relevance to our study, researchers have also conducted a study on highly diluted *Arnica montana* extract, which can be seen as a HD signal, which has shown real effects on plant physiology, including on the hormone levels of some important plant hormones, such as IAA and GA3 [16,17].

In addition to biological methods [18,19,20,21], various physicochemical or physical measurement methods [22,23,24,25] are used in HD signal demonstration research. Even if they examined a difference between an HD signal from a specified source (a tincture or some chemical) and equally processed water with no diluted material (i.e., control), they frequently found a statistically significant difference. In such HD study cases, the observed outcomes cannot be ascribed to chemical differences but rather to the HD signal itself.

Despite many published studies with a scientifically sound methodology and positive outcomes, the phenomenon of a measurable and effective HD signal is not yet widely accepted in the scientific community. The signal exhibits the ability to imprint itself into water or aqueous solutions, the capacity for subsequent transmission, and the potential to produce biological effects, with the imprint persisting in the water solution far beyond conventional physical expectations, which typically suggest lifetimes of only a few picoseconds [26].

However, a broader physical theory is not empty of ideas and general models on how to cover the observed HD phenomena. Among theoretical approaches, the work of quantum electrodynamics (QED) theorists such as Preparata, Del Giudice, Vitiello, and others appears to be the most thoroughly elaborated and convincing [27,28,29]. Their theory postulates that water is not based only on the Brownian movement of molecules and solutes, but also contains dynamically ordered water assemblies called *coherent domains*. There, electrons bound to water molecules are supposed to move in synchrony, i.e., coherently. The domains, which usually vary in diameter from one to a few hundred nanometers, constitute the so-called mesoscopic water phase, a phase between nanoscopic (molecular) and macroscopic (bulk water) [30,31,32,33,34]. Based on the theoretical framework cited above, the most likely hypothesis is that the signal is captured and retained by coherent domains, i.e., stable mesoscopic water structures. Such water assemblies have also been empirically observed through various methods, as demonstrated in experiments conducted by Konovalov and Ryzhkina [35] and Sedlák and Rak [36].

The QED theory of coherent water domains that tries to explain HD phenomena states that the encoding and retention of molecular information closely resembles the process observed in materials containing domains with magnetic moments [37,38]. According to this theory, coherent domains are characterized by ferroelectric properties arising from dynamically ordered cold vortices, thereby endowing them with the ability to function as enduring repositories for informational storage [39].

Given the above QED framework for a basic understanding and explaining the HD phenomena, we also take it as a foundation for understanding the phenomenon of HD signal transfer that transpires beyond any direct contact between two aqueous solutions. It means that the transmission should extend beyond any dynamically organized previously mentioned water structures. The transfer phenomenon has been demonstrated multiple times. Independent research teams have shown that the HD signal can be transmitted from one liquid (the donor) to another (the recipient) without direct material contact between them [6,40,41,42]. This suggests that the nature of the HD signal is complex and only partially related to mesoscopic liquid structures, such as coherent water domains, clusters, nanobubbles, nano-associates, and similar entities. It appears that at least one aspect of the signal can go beyond these water structures and be transmitted through media such as glass, air, or magnetic fields. However, in line with Ockham’s razor, we propose that the HD signal is a single entity, regardless of whether it resides in ordered water domains, glass, or air. It likely represents a specific, coherently oscillating energy or field structure interacting with mesoscopic structures within liquids as its dipolar material anchorage. When certain physical conditions (such as vigorous shaking or knocking) are met, the signal may leave its temporary location within the liquid by radiating from it and can be transferred to, absorbed by, and stored in another liquid, being physically and chemically separate [43,44,45,46]. The HD signal is generally presumed to have an electromagnetic basis [1,42,47,48]. However, while it may manifest as coherent oscillations in mesoscopic water domains, outside of water, potentially, it could represent a quantum phenomenon or involve a yet unknown quantum field or the field of quasi-particles. For instance, Kernbach proposes spin transfer [49,50], while some other authors assume the Aharonov–Bohm effect to be at work and link this to coherent water domains [51]. A promising assumption offering a fresh insight into the HD signal transfer phenomenon may reside in the so-called Zhadin’s effect when coupled with the ion cyclotron resonance (ICR) theory, started by Liboff [52,53] in the framework of bioelectromagnetics. It was followed by Zhadin and the group of Italian physicists (Preparata, Del Giudice, Giuliani, etc., [54,55]), who have deepened and refined the theory by rooting it in the already mentioned QED. ICR theory and the Zhadin effect, both corroborate the QED theory of coherent domains while at the same time offering an explanation of the HD signal transfer phenomenon. Following this line, we can, at least in principle, scientifically comprehend and elucidate HD phenomena. This encompasses phenomena associated within liquids, as previously discussed in [6], as well as those related to HD signal transfer [8].

Of course, it is important whether these theoretical assumptions are also amenable to empirical validation and the detection at the level of statistical significance. The validation is important both for a more comprehensive and in-depth understanding of the phenomenon itself, as well as for verifying and further developing and refining the theoretical explanation.

In our previous publication [6], we provided a detailed exposition of the justification and framework for employing four distinct research methodologies: electrical conductivity (χ), pH measurement, oxidation reduction potential (ORP), and UV/VIS/NIR spectrometry. The primary purpose of these methods is to detect physicochemical changes resulting from the presumed existence of the HD signal transfer. The latter is expected to alter the measured water characteristics by influencing dynamically structured water domains.

As already indicated, these stabilized water domains or nanostructures pertaining to the mesoscopic liquid phase have been empirically identified by various research teams. Due to the lack of commonly accepted terminology, researchers have used different names for these water domains, including nano-associates [56], water clusters [57,58,59,60], coherent domains [26,28,29], nanobubbles [1,42], nanoparticles [47], and naneons [48].

While our previous publication extensively discussed potential detection mechanisms [6], the current paper focuses on three different HD signals regarding potencies originating from the same substance across three receiver solutions with varying pH levels (acid, neutral, alkaline) described more in detail in the Materials and Methods section.

The working hypotheses on which this study and expectations are based can be grouped into three strands. The first concerns the three different pH values of HD signal receiver solutions, where our general expectation was that otherwise identical source waters set to the appropriate pH would detect the same HD signal differently. In addition, we predict that alkaline receiver solutions would detect the HD signal the most. Namely, the general theoretical proposition regarding coherent domains says that they are negatively charged (electron donors) and could, in principle, therefore, be stabilized by cations producing alkalinity (see [61]). Here, we followed the assumption that such a signal is not only information but also, through the knocking that accompanies the transfer, brings some energy into the receiver solution, which slightly strengthens the electrically negative coherent domains, gives greater mobility to the electrons in the domains and hence a higher activity of the hydroxide ion.

The second strand concerns the three different dilutions (also potencies) of the *Arnica montana* extract named D6 (10^−6^ dilution), C30 (10^−60^ dilution) and C200 (10^−400^ dilution). Here, we expected that the potency D6 would have a mixed signal transfer effect due to (a) the still-existing substance (diluted only a million-fold), and (b) its molecular information bound to coherent water domains. The effect of C30 would only be based on the stored molecular information with no substance left and should be weaker than that of C200. However, it was difficult to predict the difference between relatively low potency (D6) and C30 because, as mentioned above, the former can mix two different sources of HD signal transmission: material and informational.

The third strand concerns the measurement methods themselves, where we mostly rely on already published results from our own and other research and, of course, on the theoretical framework. Regarding conductivity and particularly following the ICR theory, we expected that the HD signal would knock out some ions from the surface of the coherent domains, thereby slightly increasing the conductivity of the receiver solution. A similar phenomenon has been observed also in studies of HD solutions, see [37,38,39,43]. Evidence from our previous research indicates that very high HD solution combined with striking can increase the electrical voltage (tension) of water as measured by ORP [6]. This phenomenon aligns with ionization observed in exclusion zone (EZ) water near hydrophilic surfaces [62,63]. It is also consistent with the coherent water domains theory, where the HD signal may displace ions from domain surfaces, subtly altering the electrical properties of the receiver solution. Regarding the pH measurements of receiver solutions, building upon our previous investigations and the established theoretical framework, we maintained the expectation that HD signals would induce a slight ionization, resulting in a decrease in pH in acidic solutions and an increase in pH in alkaline solutions. We also anticipated that stronger HD signals, leading to higher ion dissociation, would cause a more significant deviation from neutral pH. As outlined in a review by Yinnon, the use of UV/VIS/NIR spectrometry to analyze serially diluted and vigorously shaken solutions is a reliable indicator of their ordered states [64]. In this context, the identification of various wavelength band maxima is documented (see [7] for further information). Furthermore, within exclusion zone (EZ) water research, a consistent observation of a broad peak absorption at 270 nm has been reported [65,66]. In specially treated and presumably highly ordered MiliQ water, a UV absorption band (around 300 nm) was found [67]. Theoretical propositions suggest that coherent domains, as described within the QED framework, are likely responsible for the universally observed ultraviolet (UV) absorption in dynamically structured HD water solutions [64]. Our prediction was, therefore, that the HD signal will increase absorption at least in the UV spectral range.

## 2. Results

The HD signal transfer phenomenon was investigated for three dilutions (potencies): D6, C30, and C200, in comparison to the control. The HD signal was evaluated after a 24 h exposure period in three distinct receiver solutions: neutral, acidic, and alkaline.

### 2.1. UHD Signal Transfer Detection in Neutral Receiver Solutions

Table 1 presents the Cohen’s D effect sizes for the HD signal transfer in neutral receiver solutions, indicating the relative absorption across the ultraviolet (UV), visible (VIS), and near-infrared (NIR) spectral bands. Regarding statistical significance, only the HD C200 signal in the infrared spectral band shows a noticeable difference. Considering the standardized effect, we can observe that D6 generally shows negative differences compared to the control, with the largest effects observed in the shorter spectrum wavelengths. In contrast, C30 and C200 show positive effects (higher absorption), with C30 having a stronger impact in the longer wavelengths and C200 in the shorter wavelengths.

Table 2 presents Cohen’s D effect size values of the HD signal transfer in a neutral receiver solution, measured using physicochemical methods, along with their statistical significance. Consistent with the UV/VIS/NIR spectroscopy results, statistical data analysis indicates that only the HD C200 signal demonstrates statistical significance in pH measurements (highlighted in red). It is noteworthy that the effect size of D6 for conductivity is opposite to that of C200. Regarding the pH parameter, all three dilutions (D6, C30, and C200) show that the effect size increases, becoming more pronounced from D6, the lowest, to C200, the highest. This indicates a consistent alkaline effect that intensifies with higher potencies.

**Figure 1:**
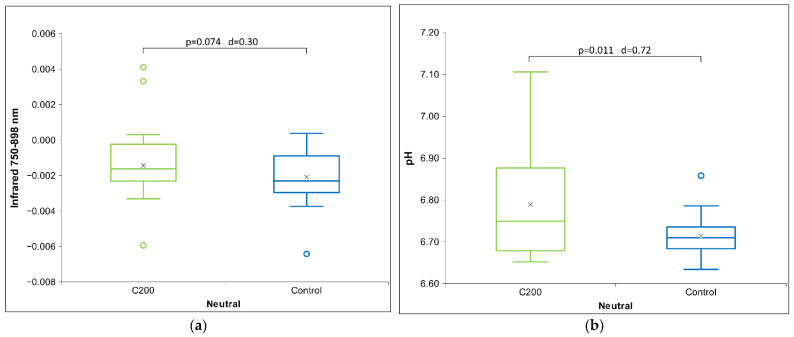
Box plot presenting measurements with significance (*p*-value and d-value) shown. (**a**) UV/VIS/NIR measurements showing relative absorption at 750–898 nm for neutral receiver solution (*N* = 18) for HD signal C200. (**b**) ORP measurements for neutral receiver solution (*N* = 18) exposed to HD signal C200. The box plots include the median and quartiles, with circles representing outliers and an ‘x’ mark indicating the mean.

From the tables above (see Table 1 and Table 2), it can be seen that for the neutral receiver solution, statistical differences are only observed for the C200 HD signal in the NIR band spectroscopy (Figure 1a), where we see that the C200 signal has a higher absorption compared to the control signal, and in pH (Figure 1b), where pH has increased statistically significantly for the C200 signal compared to the control signal.

### 2.2. UHD Signal Transfer Detection in Acidic Receiver Solutions

**Figure 2:**
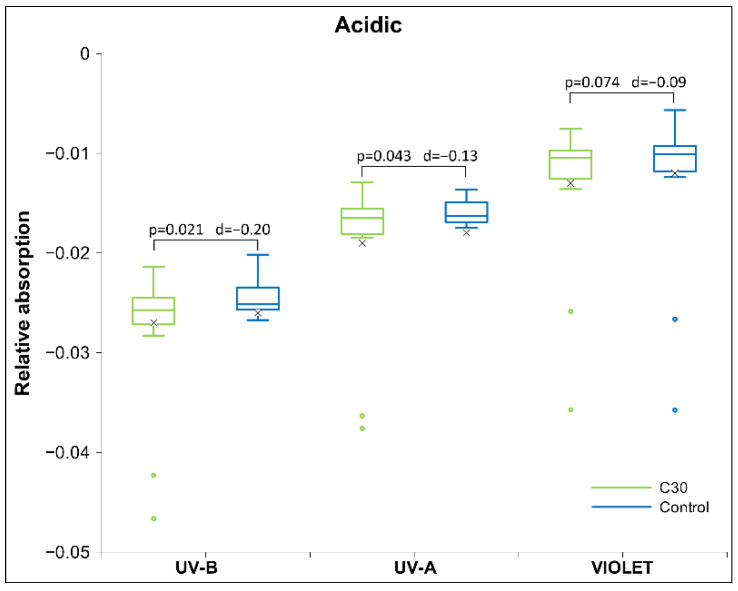
Box plot of UV/VIS/NIR measurements presenting relative absorption at UV-B, UV-A and violet bands, the median and quartiles, with circles representing outliers and an ‘x’ mark indicating the mean, for acidic receiver solution (*N* = 18) for HD signal C30. Significance (*p*-values and d-values) are presented, too.

Table 3 presents the magnitudes of Cohen’s D effects for HD signal transfer in the receiver’s acidic solution. It shows the relative absorption in the ultraviolet (UV), visible (VIS), and near-infrared (NIR) spectra, along with their statistical significance. The results show significant differences in the UV bands for the C200 signal in the UV-B and the C30 signal in both UV-B and UV-A bands (Figure 2) and no statistical difference for the D6.

As shown in Table 3 above, the acidic receiver solution C30 signal shows a significant difference, specifically for UV-B and UV-A, and a trend for violet. In all three cases, the absorbance is lower for the C30 signal vs. the control signal. The significance decreases with increasing spectral bands, as shown in Figure 2 below.

**Figure 3:**
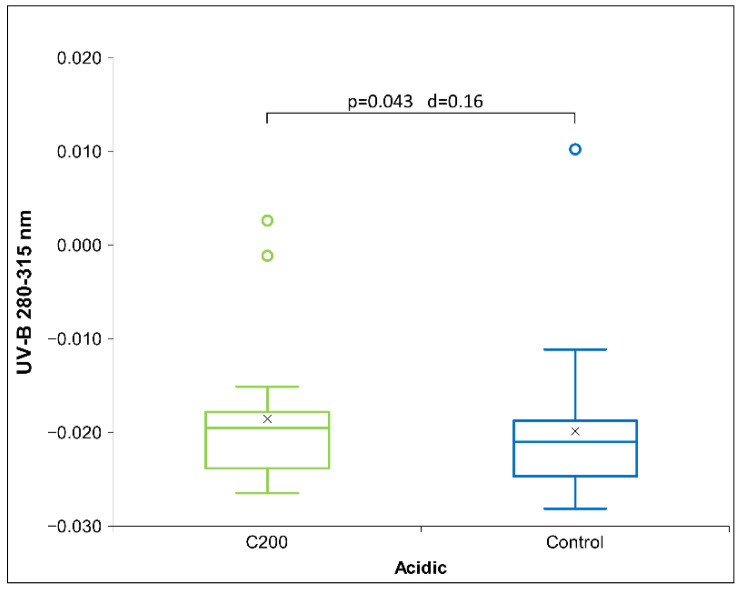
Box plot of UV-B measurements presenting relative absorption at 280–315 nm, the median and quartiles, with circles representing outliers and an ‘x’ mark indicating the mean, for the acidic receiver solution (*N* = 18) exposed to HD signal C200. Significance (*p*-value and d-value) is presented, too.

Statistical significance in the UV-B band was also detected for the HD C200 signal (Figure 3). However, the latter showed a higher absorption vs. the control signal, while the C30 signal showed a lower absorption rate (Figure 2).

Table 4 presents the values of Cohen’s D effect sizes for the HD signal transfer in acidic receiver solutions, as measured by physicochemical methods. Only the HD D6 signal demonstrates a statistical trend and a relatively high effect size for pH measurements.

**Figure 4:**
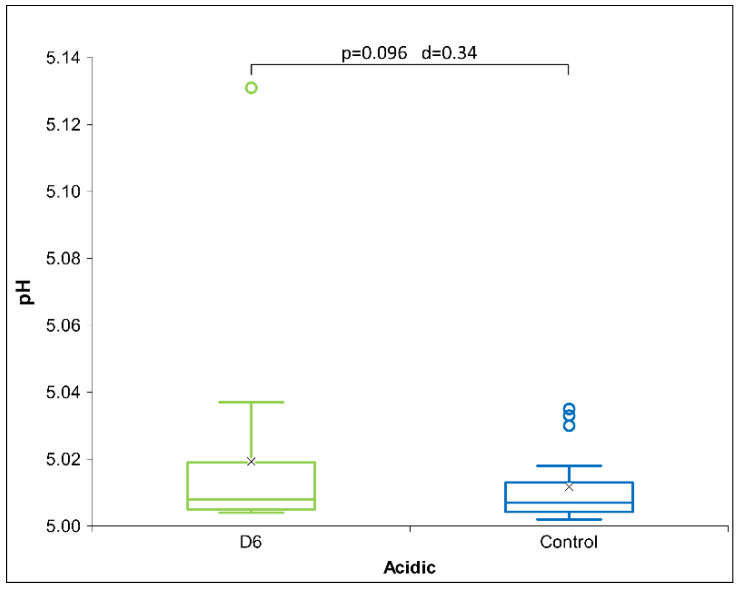
Box plot of pH measurements presenting the median and quartiles, with circles representing outliers and an ‘x’ mark indicating the mean, for acidic receiver solution (*N* = 18) exposed to HD signal D6. Significance (*p*-value and d-value) is presented, too.

The acidic receiver solution exhibits no statistically significant differences in the physicochemical methods for HD signals C30 and C200 (see Table 4). Disregarding statistical insignificance, we may say that D6 again shows a negative influence in conductivity (lowering it) and the opposite trend in pH among potencies: the highest difference for D6 and the lowest for C200. However, a statistical trend is observed with the D6 signal in the pH measurements (Figure 4), indicating a shift towards the neutral pH.

### 2.3. HD Signal Transfer Detection in Alkaline Receiver Solutions

**Figure 5:**
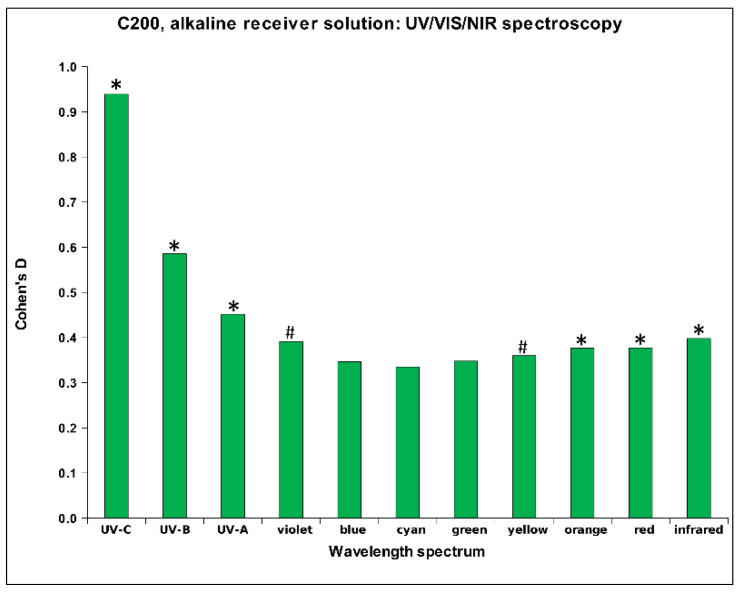
Relative mean values of Cohen’s D for the UV/VIS/NIR measurements of alkaline receiver solution exposed to C200 HD signal, (*N* = 18). Differences were considered statistically significant with *p* < 0.09 (#; a trend), *p* < 0.05 (*).

Table 5 presents the magnitudes of Cohen’s D for the HD signal transfer in the receiver’s alkaline solution, indicating the relative absorption in the ultraviolet (UV), visible (VIS), and near-infrared (NIR) spectral bands, along with their statistical significance. The results demonstrate statistically significant differences for the HD C200 signal for a large part of the monitored spectrum: from UV-C to UV-A bands and from orange to infrared bands (see also Figure 5). Additionally, a statistical trend can be observed from violet and yellow bands. As shown in Table 5, a statistical trend is observed in the UV-C spectral band, where the C30 signal exhibits lower absorption vs. the control (see Supplementary Materials Figure S1), and not statistically significant for the D6 signal. A similar trend was previously observed for acidic water (see Table 3), where both lower absorption of the C30 signal vs. the control and a statistically significant difference were obtained (see Figure 2).

As can already be seen from Table 5 above, the HD C200 signal shows a very consistent positive effect on the absorption rate in the alkaline receiver solution (see Figure 5), supporting the effect of the other two solutions.

**Figure 6:**
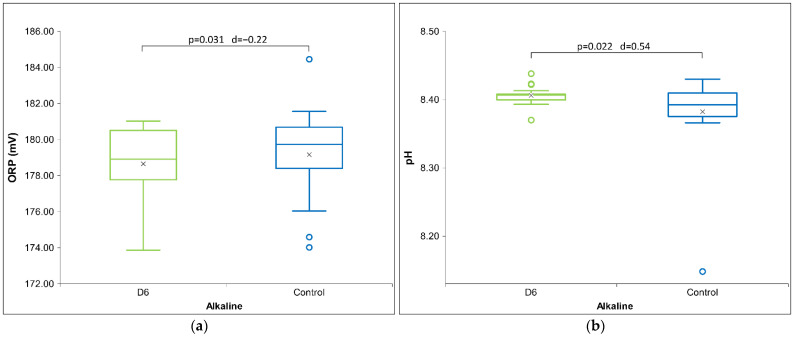
Box plot presenting measurements with significance (*p*-value and d-value) shown. (**a**) ORP measurements for alkaline receiver solution (*N* = 18) exposed for HD signal D6. (**b**) pH measurements for alkaline receiver solution (*N* = 18) exposed to HD signal D6. The box plots include the median and quartiles, with circles representing outliers and an ‘x’ mark indicating the mean.

**Figure 7:**
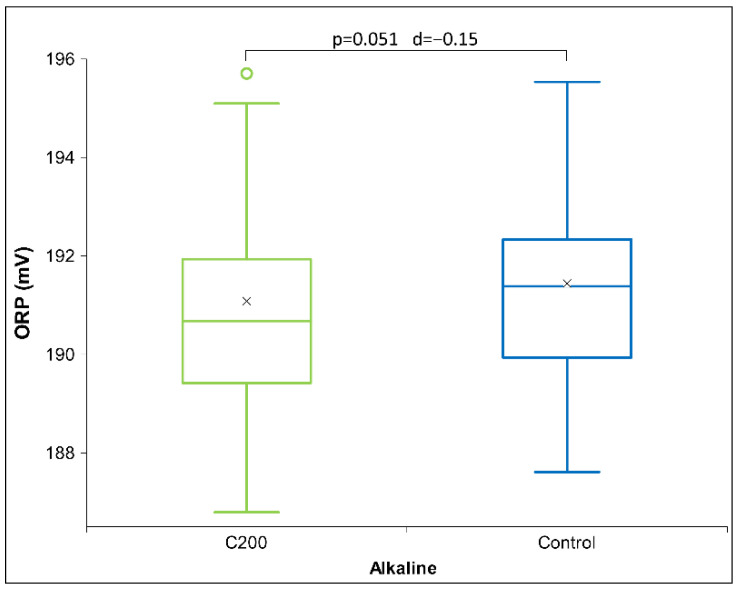
Box plot presenting measurements with significance (*p*-value and d-value) shown. ORP measurements for alkaline receiver solution (*N* = 18) exposed to HD signal C200. The box plots include the median and quartiles, with circles representing outliers and an ‘x’ mark indicating the mean.

Table 6 presents Cohen’s D effect sizes for HD signal transfer in an alkaline receiver solution, as measured by physicochemical methods. The results show that contrary to the previous two solutions, HD D6 signal measurements indicate a strong impact on the receiver solutions that exhibits especially with pH and ORP measurements (Figure 6). Regarding the pH measurements through all three pH solutions, the results indicate an alkaline effect (raising the pH) that is maximal with the alkaline solutions. Furthermore, the HD C200 signal demonstrates statistical significance in ORP (Figure 7) and a trend in conductivity measurements (see Supplementary Materials Figure S2). No differences are observed with the C30 HD signal. Overall, the results indicate that both extreme signals (D6 and C200) lowered the voltage (ORP) and electrical conductivity for the C200 signal.

Figure 6a below shows that the D6 signal is statistically significant at the ORP measurement, as the voltage is reduced compared to the control. In terms of pH measurement (see Figure 6b), the results indicate an alkaline effect (increase in pH), which is also statistically significant.

From Figure 7 below, it can be observed that the voltage of the C200 signal decreased, consistent with the observations for the D6 signal (Figure 6a), which also exhibited a statistical trend. Similarly, the electrical conductivity decreased vs. the control signal (see Supplementary Figure S2), showing a statistical trend.

## 3. Discussion

In this study, we conducted further systematic, even if still somewhat pilot, research of the HD signal transfer. If in the previous two studies, we examined the effectiveness of such transfers and its characteristics concerning exposure time and distance [6,7]. In the present study, which involves a different originating HD source substance, we expanded the investigation to include three different pH levels and potencies. This extension aims to provide insight into how the signal interacts with the ionic environment of water. In addition, we also performed much more detailed UV/VIS/NIR spectroscopic analyses.

To evaluate the effects of HD signal transfer on various parameters and conditions in more depth, we integrated data from all HD signal transfer experiments in different ways, such as across all three potencies and for individual potencies across all three receiver solutions. To ensure all data were comparable for statistical analysis, we normalized them.

**Figure 8:**
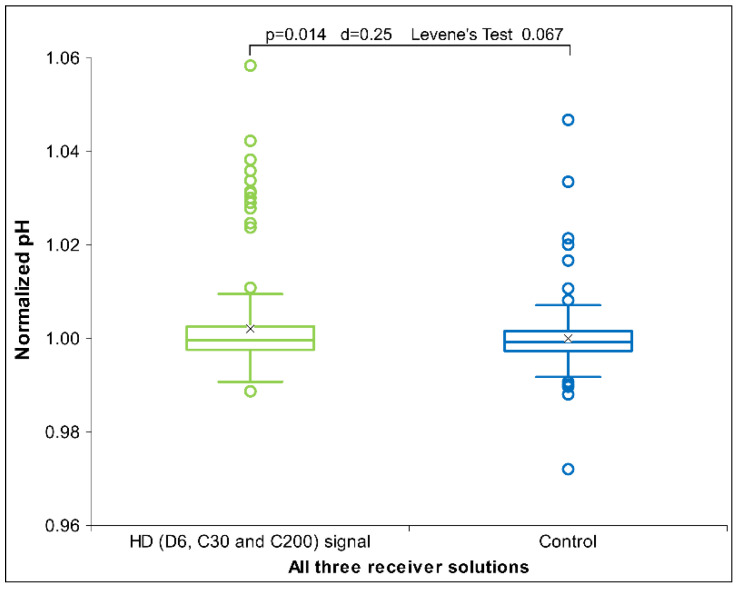
Box plot of normalized pH measurements for the HD signals (D6, C30 and C200), presenting the median and quartiles, with circles representing outliers and an ‘x’ mark indicating the mean, for all three receiver solutions (neutral, acidic, and alkaline) (*N* = 162). Significance analyses (*p*-value, d-value and Levene’s Test) differences are presented, too.

Regarding our assumptions presented in the Introduction, we will first examine the outcomes concerning the effects on the pH of different solutions. The assumption was that the HD signal transfer would, in general, slightly increase ionization, which would show as distancing from the neutral pH. However, we notice a predominantly alkaline effect, i.e., raising the pH of receiver solutions (see Supplementary Materials Table S1 for qualitative illustration). The alkaline water revealed interesting pH variations across different HD signal influences. While the lower potency (the signal of D6) demonstrated an alkaline effect, the highest potency (C200) showed a neutralizing or slightly acidic effect, and the intermediate potency (C30) showed no significant influence on pH change. When integrating all three kinds of HD signal across all three receiver solutions (neutral, acidic, and alkaline), we identified a statistically significant difference between the HD signal and the control situations (Figure 8) in the sense of raising pH (alkalinity promoting effect). A similar observation is reported by Ryzhkina et al. [68], who demonstrated in their study that specific antibody dilutions exhibited an increase in pH by approximately two units compared to corresponding dilutions of control water.

**Figure 9:**
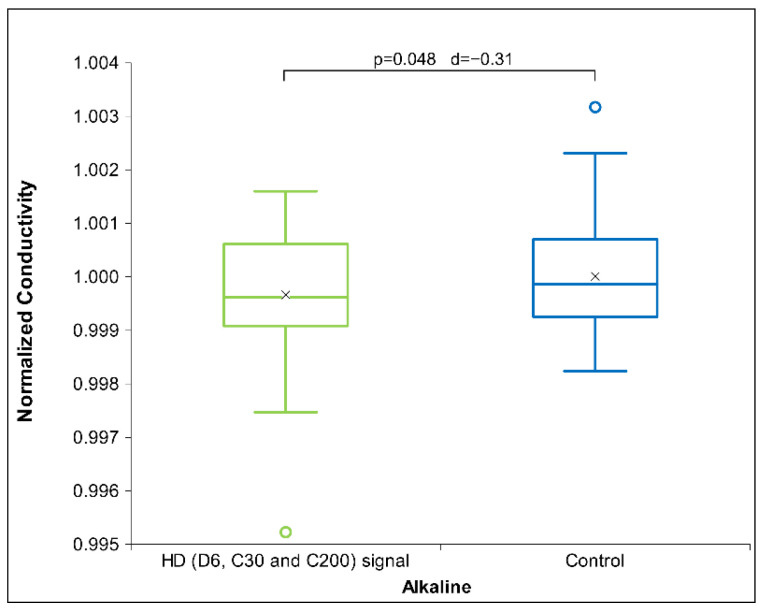
Box plot of normalized conductivity measurements for the HD signals (D6, C30, and C200), presenting the median and quartiles, with circles representing outliers and an ‘x’ mark indicating the mean, for alkaline receiver solution (*N* = 54). Significance (*p*-value and d-value) is presented, too.

Regarding effects on conductivity, the assumption in the Introduction stated that the signal should rise due to knocking out some ions, as ICR theory puts it [37,38,39,43]. However, in the used specific receiver solution we noticed the opposite effect, as qualitatively illustrated in Supplementary Materials Table S2, and presented also in Figure 9 for the alkaline solution and for all three dilutions. It appears that the solution containing some residual material in its molecular information transfer consistently slowed down ion mobility, whereas the highest potency (C200) had this effect only in the alkaline solution. In our previous studies, we observed similar behavior during overnight exposures with a different receiver solution and without activation [7]. Consequently, it seems that conductivity may be highly sensitive to various conditions of HD signal transfer, such as the type of water, the method of exposure, and possibly even the specific original substance. It is of interest that an investigation by another research group observed a similar phenomenon. Namely, exposing distilled water to low frequencies and low-intensity magnetic fields (<5 mT) resulted in a measurable decrease in water conductivity within 24 h [69].

**Figure 10:**
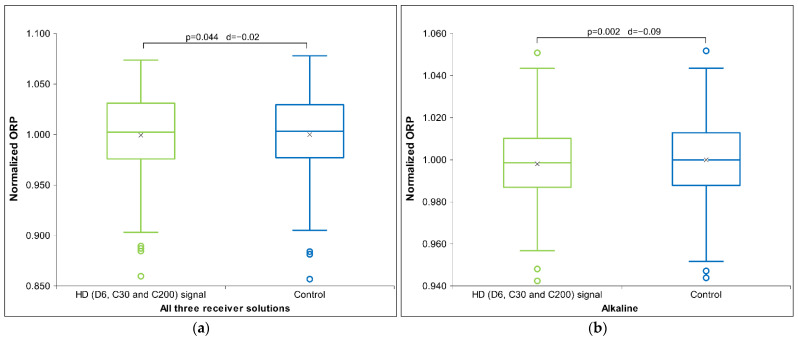
Box plot of normalized ORP measurements for the HD signals (D6, C30, and C200), presenting the median and quartiles, with circles representing outliers and an ‘x’ mark indicating the mean, for (**a**) all three receiver solutions (neutral, acidic, and alkaline) (N = 153), and (**b**) alkaline receiver solution (*N* = 51). Significances (*p*-values and d-values) are presented, too.

We had a similar expectation, expressed in the Introduction, for water voltage (as indicated by ORP measurements); however, the results showed the opposite response of our receiver solutions. Namely, through the integration of all ORP measurements, involving all three kinds of signals across all three receiver solutions (comprising 153 independent measurements), a statistically significant lower ORP value was observed (Figure 10a). As shown in Figure 10b, the effect is most pronounced in the alkaline receiver solution, with a noticeable statistical significance, as also evidenced in Table 6 or Figure 6a and Figure 7. The result for the alkaline solution closely parallels conductivity (Table 6) and strongly supports the finding that the signal transfer of the middle dilution (C30) was detected most weakly, if at all. By combining all three types of signals across the three receiver solutions, we were able to validate the observed trends and reinforce the statistical significance of the findings, ensuring that the results were not driven by any individual data set or specific condition.

Further research should investigate whether a common cause underlies the observed parallelism between conductivity and ORP in alkaline solutions. However, since the ORP results are not correlated with the pH measurements, we may assume that the effect of the HD signal on ionization is less responsible for the decrease in ORP than its effect on the dissolution of oxygen or CO_2_, which solubility, in this case, would slightly drop. Further research should dive into the underlying mechanisms of this phenomenon.

Turning to spectral analyses (see Table 1, Table 3 and Table 5), we observe consistently lower relative absorption values for acidic solutions compared to alkaline ones. For the HD signals D6 and C30, the latter even shows negative values compared to the control. The difference in the detection of the HD signal between alkaline and acidic solutions becomes even more apparent when focusing solely on the three UV bands (see Supplementary Materials Table S3 for qualitative illustration). This unexpected difference suggests an active and different involvement of hydroxide and/or hydronium ions in detecting HD signals. This phenomenon warrants further research to provide more in-depth theoretical explanations regarding the nature of HD signals.

In a previous article [7], we reported that the UV/VIS increased the absorption rate through the potentiation process compared to the potentiated pure distilled water vs. control. Interestingly, for neutral water, Cohen’s D values increase with higher potency, whether considering only the UV spectral part or the entire observed spectrum. This indicates that neutral water may be more sensitive to potency (dilution) changes than acidic or alkaline water.

**Figure 11:**
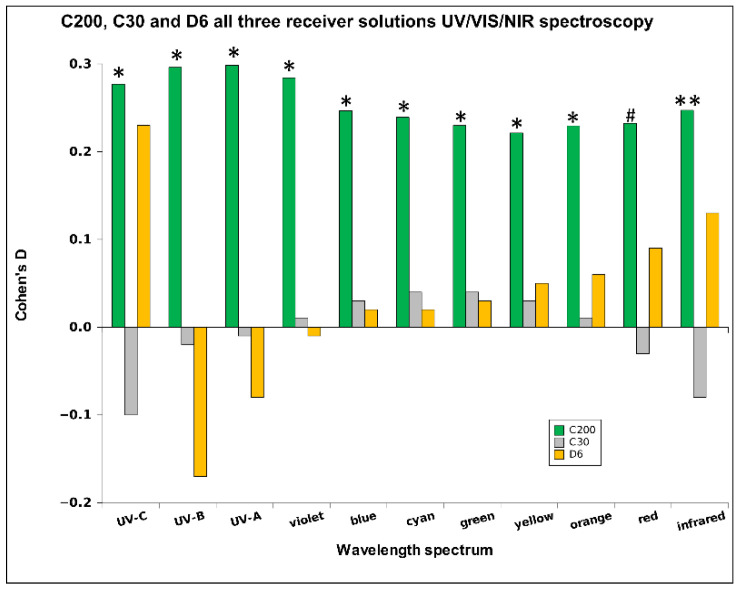
Relative mean values of Cohen’s D for the UV/VIS/NIR measurements with all three receiver solutions, (*N* = 54). Differences were considered statistically significant with *p* < 0.09 (#), *p* < 0.05 (*), *p* < 0.01 (**).

In Figure 11, we present the results of all three HD signals across all three receiver solutions per spectral band, showing also their statistical significance (*N* = 54). We can notice the strength of the C200 signal, with the most pronounced statistical significance in the infrared band. Considering also Table 1, Table 3 and Table 5 conveying more detailed effect sizes per type of receiver water, we may infer that the signal C200 consistently influenced receiver waters with the highest impact on alkaline water (Table 5). Notably, while the signal detectability through bands in alkaline water (Figure 6) exhibits a similar pattern, it becomes significantly more consistent and statistically robust when all three water types are considered together (Figure 11). The D6 signal (if we exclude the UV-C band) shows a consistently higher absorption rate by longer wavelengths. Considering the above-referenced Tables, this signal may achieve relatively high effect sizes but no statistical significance, which speaks about its high instability (variability). As per the observed spectrum, the highest signal transfer detectability (disregarding the sign of Cohen’s D) stands in the UV region, attenuates in most of the VIS region, and again increases towards the end of the VIS and in the NIR band.

**Figure 12:**
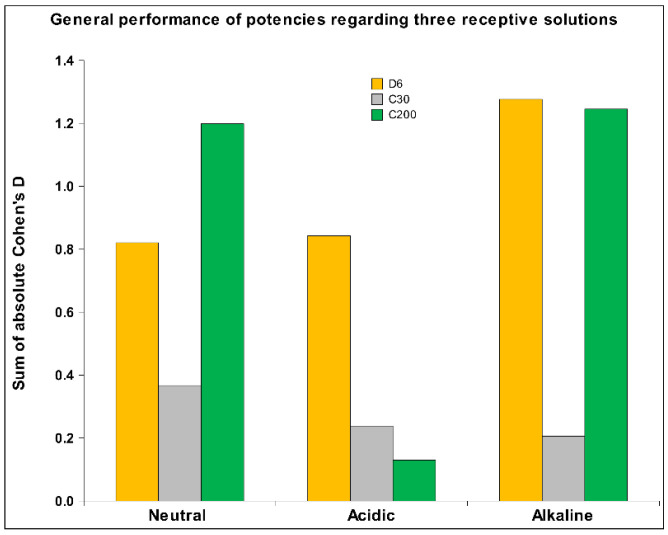
The sum of absolute Cohen’s D for each of the three HD signals is presented separately, which integrates data from all four measurement methods across the three types of receiver solutions.

Another assumption stated that regarding the difference in the pH, the solutions would detect the same HD signal differently. The results confirm this hypothesis, showing an overall similarity in the detection abilities of neutral and alkaline solutions, with a significantly lower detection ability in the acidic solution (Figure 12). However, a closer inspection of the different signals reveals a broader range of differences.

The hypothesis that the alkaline solution would generally have the best detection capabilities has also been confirmed, especially when we set aside the neutral solution, which performs only slightly lower. Comparing the performance of the acidic and alkaline solutions (Figure 12), the superior detection capabilities of the alkaline solution are evident, especially when we focus solely on physicochemical results (Table 6).

**Figure 13:**
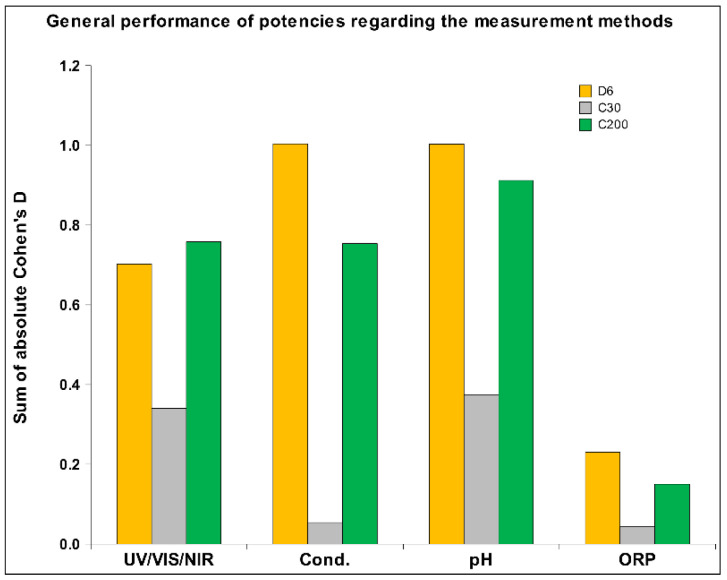
The sum of the absolute Cohen’s D for all three HD signals integrating all three kinds of receiver solutions across all four measurement methods.

Regarding the three different potencies of the *Arnica montana* extract, we postulated that the potency C200 would have a stronger impact on the receiver solution than C30, which was validated (see Figure 12). The D6 signal was expected to have a dual impact: one derived from the still detectable presence of the original substance and the other from the potency itself. It demonstrated a significantly stronger effect than C30 and even slightly outperformed the extremely highly potentized C200 (see Figure 13). The difference indicates that on one side, the substance—even if diluted—has its own impact, while on the other side, the potency level also has its influential power. As shown in Figure 13, it is evident across all four research methods that C30 performed the least well, probably because C30 does not have as high a potency as C200, and on the other hand no longer has any of the original substance as D6. We also assessed detectability across different measurement methods, where it became evident that the ORP method was the least effective, consistent with observations from our previous study [6].

## 4. Materials and Methods

### 4.1. Material and Devices

The experiment utilized *Arnica montana* brand granules with varying potencies (D6, C30, and C200) from Salvator Pharmacy (Eisenstadt, Austria), while Narayana Verlag (Kandern, Germany) provided the control granules. The granules used for the control situation were identical by form (size 3, circa 2.2 mm) and were of the same substance (Sucrose, formula: C_12_H_22_O_11_) as *Arnica montana* ones.

**Figure 14:**
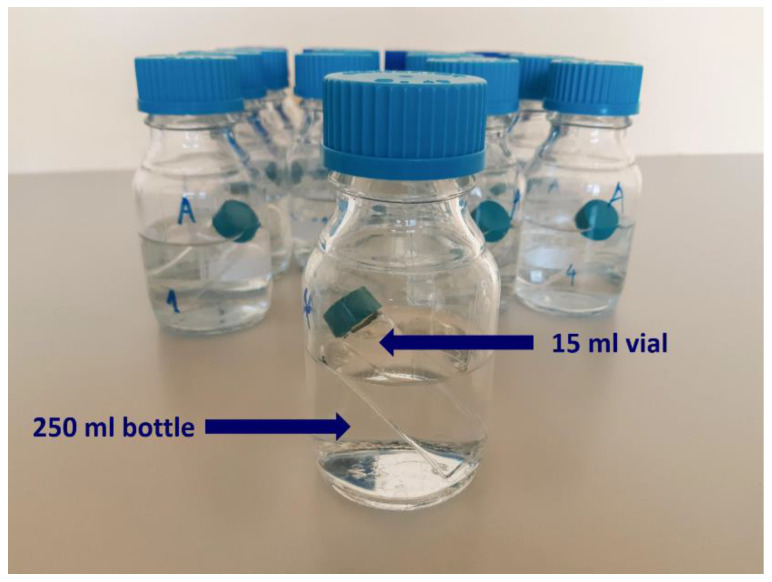
Demonstration of experimental situation. Vials with the solution of granules inserted into 250 mL glass bottles filled with receiver solution. This setup was used for both HD signal and control samples.

The HD solution was prepared by dissolving 1.2 g of granules (D6, C30, C200, and control granules) in a mixture of 93% distilled water and 7% ethanol (96%). The solution was potentized by tapping it against the palm 20 times, then pipetted into 15 mL vials. These vials were inserted into 250 mL bottles containing receiver solution (Figure 14).

Various potencies of *Arnica montana* have served as a source of non-contact (physical) HD signal transfer into the receiver solution. The receiver solution bottles (solution in three pH varieties) were exposed to two different situations:

- UHD signal situations:
  ○ D6: the 15 mL vials were placed into the 250 mL bottles containing the receiver solution with specified pH (see Section 4.1.1 for more information),
  ○ C30: same treatment,
  ○ C200: same treatment,
- Control situation (C): same treatment stemming from the control granules.

**Figure 15:**
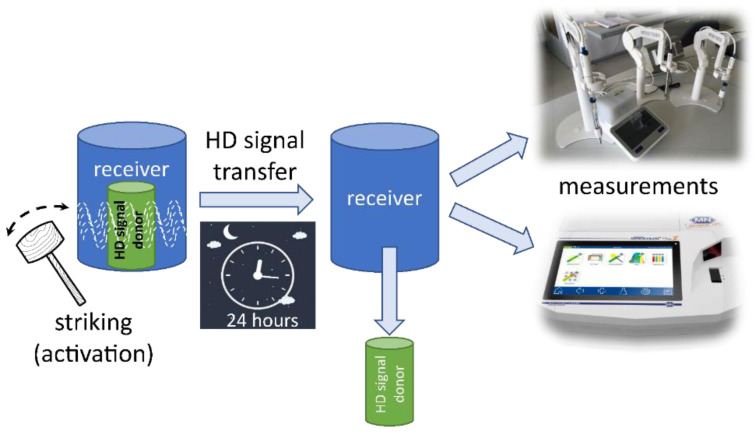
The outline of the experimental situation, showing the process applied to the HD signal. An identical procedure was conducted for the control samples for comparative analysis.

The situations were in two separate rooms to prevent any remote HD influence on the control. Consequently, one room was used for HD signal exposure, while the other was for the control. Each situation lasted for 24 h. At the outset of the experiment and for activation, each bottle, which had been previously prepared to contain vials, was struck 15 times with a wooden mallet upon its placement in the room. An outline of the experimental situation detailing the HD signal transfer is presented in Figure 15.

#### 4.1.1. Water Used for the Receiver Solutions

In our study, we chose to use commercial FIJI water as the receiver medium for HD solutions, instead of distilled water, based on several key considerations.

FIJI water has been previously utilized in scientific research exploring the impact of alleged subtle energy stemming from bioenergy therapists on water properties [70]. Since the water proved to be a good detector for these subtle impacts, we considered it useful for HD signal detection as well. The molarity of its main anion (bicarbonate) is ten times lower than in the human blood.

FIJI water contains naturally occurring minerals and electrolytes, offering a much more complex composition than distilled water. We expected that such a richer composition might offer a greater opportunity to develop different types of coherent domains that could detect a wider range of HD signals.

##### Original Fiji Water

For the fundamental receiver solution, we used FIJI Water, sourced and bottled in Viti Levu, Fiji Islands, is the top premium imported water brand in the U.S. Originating 1600 miles from the nearest continent, it is naturally filtered through volcanic rock in a rainforest, gaining minerals and electrolytes. The water emerges from a deep artesian aquifer, protected by rock layers, and is bottled directly at the source.

General mineral analysis of the FIJI Water was as follows (as of March 2023): 140 mg/L of bicarbonate, 18 mg/L of calcium, 9 mg/L of chloride, 0.3 mg/L of fluoride, 13 mg/L of magnesium, 16 mg/L of sodium, 85 mg/L of silica and 0.5 mg/L of sulfate. The total dissolved solids were 220 mg/L, and the total alkalinity was 260 mg/L. The declared pH was 7.61.

##### Further Processing of FIJI Water to Attain Three Targeted Receiver Solutions

This study aimed to detect and evaluate the effects of HD transfer on three types of solutions in terms of pH (three pH values), including mildly acidic, neutral, and mildly alkaline. We used citric acid, a common mammalian metabolite (Sigma-Aldrich, Merck KGaA, Darmstadt, Germany), to achieve the acidic targeted pH value of 5.5 and the neutral pH of around 7. Sodium hydroxide (Fluka, Honeywell Research Chemicals, Charlotte, NC, USA) was chosen to achieve alkaline pH of 8.5.

#### 4.1.2. Measurement Devices and Methods

To evaluate the impact of HD transfer on the solution, we employed the following physicochemical methods and UV/VIS/NIR to measure the specific effects of the selected HD transfer sources on the receiver solution, including electrical conductivity, pH, and ORP. We also measured the relative absorption across the UV/VIS/NIR spectrum, which was divided into the following spectral bands: UV-C (200–280 nm), UV-B (280–315 nm), UV-A (315–400 nm), violet (400–450 nm), blue (450–485 nm), cyan (485–500 nm), green (500–565 nm), yellow (565–590 nm), orange (590–625 nm), red (625–750 nm) and near-infrared (NIR) (750–898 nm). Additionally, the individual ultraviolet bands (UV-C, UV-B, and UV-A) were integrated to form a single UV spectral band (200–400 nm), while the visible spectrum (VIS) extends from 400 to 750 nm. For measurement of the physicochemical parameters, we used METTLER TOLEDO Seven Excellence S470 for pH and conductivity, and Vernier Go Direct^®^ devices (Vernier Software & Technology, Beaverton, OR, USA) for ORP. To ensure the validity of our results, we conducted simultaneous temperature measurements. This consideration was particularly important because conductivity is known to be influenced by temperature changes, but it also has some relevance to other measurement methods. Each individual measurement was conducted in a separate beaker. The measured range of accuracy (which deviates from the officially declared value and is not part of the observed, investigated, and considered measurement drift) for these three devices is as follows: ± 0.05 for pH; 2 mV for ORP. As for the conductivity measurements, the double steel pole cell with a built-in temperature probe virtually eliminates errors in the measurements.

For the UV/VIS/NIR absorption spectroscopy measurements, we used a Nanocolor^®^ UV/VIS II spectrophotometer from Macherey-Nagel (Düren, Germany), wavelength range 190–1100 nm, with a 50 mm quartz cuvette cell. The measured accuracy range here is ±1 nm (wavelength) and 0.003 of relative absorption. UV/VIS/NIR spectroscopic data can provide both qualitative and quantitative information about a given compound or molecule. Whether seeking quantitative or qualitative insights, it is crucial to employ a reference cell (zero solution) to zero the instrument for the solvent in which the compound is dissolved. Each measurement was conducted in a dedicated cuvette that was consistently used throughout the entire experiment.

### 4.2. Experimental Protocol

#### 4.2.1. Blinding

The experiment was conducted under blinded conditions, with the measurement group responsible for collecting the results and the analysts involved in data analysis unaware of the experimental situation. This approach ensured a rigorous and objective evaluation of the results, eliminating any bias or preconceptions regarding the samples. Consequently, the different experimental situations (exposures and controls, respectively) were randomly labeled with the letters A and B. So, in principle, we had labels from A1 to A18, and the same for B. Initially, an assistant prepared solution samples and filled them into bottles, labeling them A and B. Then, a researcher moved these bottles to different positions. The experimental situation pertaining to the bottle was only revealed after the statistical analysis of the results of the experimental set was finished.

#### 4.2.2. Sequential Process Management

The pouring and measuring procedures were carried out systematically and in variable order to avoid possible systematic measurement errors due to gradual (even if slight) measurement shifts over time. It is particularly important in pairwise statistical comparisons, where a significant difference can only be obtained due to a gradual shift in the measurement values over time. The pouring of the receiver solutions followed a specific order: A1, B1, A2, B2, and so on. However, the physicochemical parameters measurements followed the variable order: A1, B1, B2, A2, A3, B3, and so forth. The UV/VIS/NIR measurements were conducted using the same variable-order method, following the sequence A1, B1, zero solution, B2, A2, zero solution, A3, B3, zero solution, and so on.

#### 4.2.3. Measurement Protocol

For the physicochemical measurements, the solutions from the bottles were poured into beakers prior to measurements. Each physicochemical parameter (conductivity, pH, ORP) and temperature was measured in its own separate beaker using the variable order measurement method described above.

For the UV/VIS/NIR absorption measurements, the solution was poured into a 50 mm quartz cuvette. All solutions (control included) were diluted to a 50% ethanol solution and before being poured into the cuvette, subjected to 15 taps on the vial glass using an automated tapping device at a frequency of 4 Hz. The reference cuvette was not subjected to tapping.

### 4.3. Statistical Analysis of the Results

For estimating statistical significance, we applied appropriate tests regarding normality and sample size. The data sets were subjected to either a *t*-test (normal distribution) or a Wilcoxon signed-rank test. When integrating the outcomes of more experiments, we used the Sign test for statistical evaluation. All tests were performed via pairwise comparison.

Statistical data analysis was performed using XLSTAT statistical software (XLSTAT PREMIUM-version 2022.3.1, build version 16.0.18324) for Excel. For basic statistical parameters of data sets, we calculated the average, standard deviation, standard error, and normality with the Shapiro–Wilcoxon test. To estimate statistical significance in the data variation, we used Levene’s test (based on median) or F-test for differences in variance.

To compare the impacts measured by different measuring methods, we calculated the standardized effect sizes (Cohen’s D values) and presented them in the Results. They express the difference between HD signal detection and control.

Differences are considered statistically significant with *p* < 0.05, or if 0.05 < *p* < 0.1 and the absolute Cohen’s D value is higher than 0.5. A statistical trend is considered any result of a statistical analysis that shows 0.05 < *p* < 0.1 and an absolute Cohen’s D between 0.3 and 0.5.

#### Normalization in Multi-Experiment Evaluation

To identify the broader effects of HD signal transfer, we conducted a statistical analysis that included various experimental situations, as discussed in the Discussion. Given the differing mean values, we normalized the results to ensure comparability and minimize statistical noise.

Normalization was performed through two methods. In the first approach, we se-lected a reference data set along with its control group (designated as C1). Afterward, we calculated the difference between the average control value of C1 and that of sub-sequent sets (C2, C3, etc.) and algebraically adjusted all values within these sets by this difference. This method was applied to the UV/VIS/NIR spectrum. In the second ap-proach, we normalized the values within each data set by dividing them by the aver-age control value of their respective set, which was applied to the physicochemical measurements.

## 5. Conclusions

In conclusion, the present study we employed a slightly more rigorous protocol than in the previous ones, utilized a different starting substance, and different receiver solutions tuned to three different pH values: mildly alkaline, neutral, and mildly acidic. Also, in this modified experimental situation, we were able to confirm the effect of the physical (mechanical) transfer of the HD signal from the donor to the receiver solution. This signal was measurable at statistically significant levels across all four measurement methods, reinforcing our general expectation regarding the robustness of the HD transfer phenomenon.

The results partially confirmed our more specific assumptions; however, we also encountered unexpected phenomena. From this more specified view, the following conclusions can be drawn.

First, we observe a general effect of raising alkalinity of the exposed liquids that emerge if we integrate all three pH-types of receiver solutions and all three potencies of HD signals (see Figure 8).

Second, different potencies may exert different influential powers, and this effect is not linear as regards the magnitude of the potency (see Figure 11, Figure 12 and Figure 13). This can also be observed in UV/VIS/NIR spectroscopy, as we see a relatively strong influence of the high-potency HD signal (C200) in the direction of increased relative absorption throughout the spectrum, a negligible impact of the C30 potency and almost monotonically increasing influence (from negative to positive values) of the low-potency (D6).

Third, Given that statistically significant comparisons revealed an overall reduction in electrical conductivity and voltage (ORP), we conclude that the HD signal generally influences the receiver solutions by slightly reducing their (auto)ionization (see Figure 9 and Figure 10).

Fourth, regarding the most conspicuous effects on the alkaline receiver water (see Table 5 as compared to Table 1 and Table 3; Figure 5, Figure 6, Figure 7, Figure 9 and Figure 10b), we can assume that hydroxide ions with clusters (coherent domains) around them may be specifically sensitive to HD signal transfer impact.

The overall findings of the study offer valuable new insights and suggest innovative approaches for further research, particularly in detecting the HD signal in solutions with varying pH levels, focusing on interactions with hydroxide and hydronium ions.

## Figures and Tables

**Table 1:** Standardized effect sizes (Cohen’s D; see Section 2.3 for further detail) of the relative absorption rate across the UV/VIS/NIR spectrum for the HD signal impacted samples in neutral receiver solution. The sample size (N) for each spectral band is 18.

Neutral Receiver Solutions				
Spectral Band		D6	C30	C200
UV-C	200–280	0.48	−0.06	0.22
UV-B	280–315	−0.39	0.01	0.23
UV-A	315–400	−0.34	0.04	0.24
Violet	400–450	−0.28	0.05	0.30
Blue	450–485	−0.25	0.08	0.27
Cyan	485–500	−0.26	0.10	0.25
Green	500–565	−0.25	0.14	0.18
Yellow	565–590	−0.26	0.19	0.06
Orange	590–625	−0.26	0.20	0.05
Red	625–750	−0.15	0.20	0.20
Infrared	750–898	−0.11	0.18	0.30

Statistical significance value (*p* < 0.05) is highlighted in red.

**Table 2:** Standardized effect sizes for the HD samples in neutral receiver solution were measured using three different physicochemical methods. For each measurement, *N* = 18.

Neutral Receiver Solutions			
	D6	C30	C200
Conductivity	−0.39	−0.02	0.27
ORP	0.02	0.00	0.00
pH	0.13	0.23	0.72

Statistical significance value (*p* < 0.05) is highlighted in red.

**Table 3:** Standardized effect sizes of the relative absorption rate across the UV/VIS/NIR spectrum for the HD signal impacted samples in acidic receiver solution. For each spectral band, *N* = 18.

Acidic Receiver Solutions				
Spectral Band		D6	C30	C200
UV-C	200–280	−0.34	−0.20	0.12
UV-B	280–315	−0.33	−0.20	0.16
UV-A	315–400	−0.32	−0.13	0.14
Violet	400–450	−0.24	−0.09	0.09
Blue	450–485	−0.21	−0.08	0.05
Cyan	485–500	−0.20	−0.08	0.08
Green	500–565	−0.23	−0.09	0.08
Yellow	565–590	−0.27	−0.09	0.15
Orange	590–625	−0.24	−0.09	0.16
Red	625–750	−0.20	−0.06	−0.02
Infrared	750–898	−0.18	−0.07	−0.08

Statistical significance values (*p* < 0.05) are highlighted in red, and statistical trend in pink.

**Table 4:** Standardized effect sizes for the HD samples in acidic receiver solution were measured using three different physicochemical methods. For each measurement, *N* = 18.

Acidic Receiver Solutions			
	D6	C30	C200
Conductivity	−0.26	0.00	0.02
ORP	0.00	−0.01	0.00
pH	0.34	0.12	−0.01

Statistical trend is highlighted in pink.

**Table 5:** Standardized effect sizes of the relative absorption rate across the UV/VIS/NIR spectrum for the HD signal impacted samples in alkaline receiver solution. For each spectral band, *N* = 18.

Alkaline Receiver Solutions				
Spectral Band		D6	C30	C200
UV-C	200–280	0.47	−0.35	0.94
UV-B	280–315	0.04	0.07	0.59
UV-A	315–400	0.08	0.02	0.45
Violet	400–450	0.12	0.06	0.39
Blue	450–485	0.12	0.08	0.35
Cyan	485–500	0.12	0.09	0.34
Green	500–565	0.14	0.06	0.35
Yellow	565–590	0.19	−0.03	0.36
Orange	590–625	0.21	−0.07	0.38
Red	625–750	0.20	−0.19	0.38
Infrared	750–898	0.25	−0.30	0.40

Statistical significance values (*p* < 0.05) are highlighted in red, and statistical trend in pink.

**Table 6:** Standardized effect sizes for the HD samples in alkaline receiver solution, were measured using three different physicochemical methods. For each measurement, *N* = 18.

Alkaline Receiver Solutions			
	D6	C30	C200
Conductivity	−0.35	−0.03	−0.47
ORP	−0.21	−0.03	−0.15
pH	0.54	0.02	−0.18

Statistical significance values (*p* < 0.05) are highlighted in red.

## Data Availability

Research data are available from the BION Institute upon special request.

## Supplementary Materials

The following supporting information can be downloaded at: https://www.mdpi.com/article/10.3390/ijms26031115/s1.

## References

[1] Foletti A., Ledda M., Lolli M.G., Grimaldi S., Lisi A. (2017). Electromagnetic information transfer through aqueous system. Electromagn. Biol. Med..

[2] Foletti A., Ledda M., D’Emilia E., Grimaldi S., Lisi A. (2012). Experimental finding on the electromagnetic information transfer of specific molecular signals mediated through the aqueous system on two human cellular models. J. Altern. Complement. Med..

[3] Nagai M.Y.D.d.O., Mohammad S.N., Pinto A.A.G., Coimbra E.N., Peres G.B., Suffredini I.B., Bernardi M.M., Tournier A.L., Jerman I., Cartwright S.J. (2023). Highly diluted glyphosate mitigates its effects on Artemia Salina: Physicochemical implications. Int. J. Mol. Sci..

[4] Galeazzi B.V., Manzalini A., Cartwright S., Bonamin L., Medeiros N., Suffredini I. (2022). Silicea Terra 200cH evaluated by two different spectroscopy methods: A pilot study. Int. J. High Dilution Res..

[5] Borghini F., Dinelli G., Marotti I., Trebbi G., Borghini G., Betti L. (2021). Electromagnetic information transfer (EMIT) by ultra high diluted (UHD) solutions: The suggestive hypothesis of an epigenetic action. Int. J. High Dilution Res..

[6] Jerman I., Ogrizek L., Jan L., Periček Krapež V. (2023). Physicochemical study of the molecular signal transfer of ultra-high diluted antibodies to interferon gamma. Int. J. Mol. Sci..

[7] Jerman I., Ogrizek L., Krapež V.P., Jan L. (2024). Molecular signal transfer of highly diluted antibodies to interferon-gamma regarding kind, time, and distance of exposition. Int. J. Mol. Sci..

[8] Penkov N. (2021). Antibodies processed using high dilution technology distantly change structural properties of IFNγ aqueous solution. Pharmaceutics.

[9] Novikov V.V., Yablokova E.V. (2022). Interaction between highly diluted samples, protein solutions and water in a controlled magnetic field. Appl. Sci..

[10] Fesenko E.E., Yablokova E.V., Novikov V.V. (2024). Weak magnetic fields regulate the ability of high dilutions of water to enhance ROS production by neutrophils. Appl. Sci..

[11] Parafiniuk A., Kromer K., Fleszar M.G., Kreitschitz A., Wiśniewski J., Gamian A. (2023). Localization of sesquiterpene lactones biosynthesis in flowers of *Arnica taxa*. Molecules.

[12] Röhrl J., Piqué-Borràs M.-R., Jaklin M., Werner M., Werz O., Josef H., Hölz H., Ammendola A., Künstle G. (2023). Anti-inflammatory activities of *Arnica montana* planta tota versus flower extracts: Analytical, in vitro and in vivo mouse paw oedema model studies. Plants.

[13] Kriplani P., Guarve K., Baghael U.S. (2017). *Arnica montana* L.—A plant of healing: Review. J. Pharm. Pharmacol..

[14] Ernst E., Pittler M. (1998). Efficacy of homeopathic arnica: A systematic review of placebo-controlled clinical trials. Arch. Surg..

[15] Melnyk N., Vlasova I., Skowrońska W., Bazylko A., Piwowarski J.P., Granica S. (2022). Current knowledge on interactions of plant materials traditionally used in skin diseases in Poland and Ukraine with human skin microbiota. Int. J. Mol. Sci..

[16] Mirzajani F., Mirzajani F., Aelaei M. (2021). Study of germination efficiency and temperature/drowning resistance in some ornamental plants treated with ultra high dilute compounds. Int. J. High Dilution Res..

[17] Hosseinian S., Maute C., Rahimi F., Maute C., Hamedi M., Mirzajani F. (2020). The influence of ultra-high diluted compounds on the growth and the metabolites of *Oriza sativa* L.. Int. J. High Dilution Res..

[18] Endler P., Thieves K., Reich C., Matthiessen P., Bonamin L., Scherr C., Baumgartner S. (2010). Repetitions of fundamental research models for homeopathically prepared dilutions beyond 10^−23^: A bibliometric study. Homeopathy.

[19] Bonamin L.V. (2019). Homeopathy and experimental infections: In vivo and in vitro experiments with bacteria, fungi and protozoan. La Rev. D’homéopathie.

[20] Pinto A.A.G., Nagai M.Y.d.O., Coimbra E.N., Mohammad S.N., Silva J.S., Ancken A.V., Pinto S.A.G., Aguiar M.S., Dutra-Correa M., Hortellani M.A. (2021). Bioresilience to mercury chloride of the brine shrimp artemia salina after treatment with homeopathic mercurius corrosivus. Homeopathy.

[21] Nagai M.Y.O., Von Ancken A.C.B., Bonamin L.V. (2022). Effects of highly diluted substances on aquatic animals: A Review. Water—A Multidiscip. Res. J..

[22] Klein S.D., Würtenberger S., Wolf U., Baumgartner S., Tournier A. (2018). Physicochemical investigations of homeopathic preparations: A systematic review and bibliometric analysis—Part 1. J. Altern. Complement. Med..

[23] Tournier A., Klein S.D., Würtenberger S., Wolf U., Baumgartner S. (2019). Physicochemical investigations of homeopathic preparations: A systematic review and bibliometric Analysis—Part 2. J. Altern. Complement. Med..

[24] Bonamin L.V., Pedro R.R.P., Mota H.M.G., Aguiar M.S.C., Pinto S.A.G., de Souza J., de Oliveira L.H.S., Aparicio A.C., Peres G.B., Suffredini I. (2020). Characterization of antimonium crudum activity using solvatochromic dyes. Homeopathy.

[25] Tournier A., Würtenberger S., Klein S.D., Baumgartner S. (2021). Physicochemical investigations of homeopathic preparations: A systematic review and bibliometric Analysis—Part 3. J. Altern. Complement. Med..

[26] Van Der Post S.T., Hsieh C.-S., Okuno M., Nagata Y., Bakker H.J., Bonn M., Hunger J. (2015). Strong frequency dependence of vibrational relaxation in bulk and surface water reveals sub-picosecond structural heterogeneity. Nat. Commun..

[27] Del Giudice E., Preparata G., Vitiello G. (1988). Water as a free electric dipole laser. Phys. Rev. Lett..

[28] Arani R., Bono I., Del Giudice E.D., Preparata G. (1995). Qed coherence and the thermodynamics of water. Int. J. Mod. Phys. B.

[29] Bono I., Del Giudice E., Gamberale L., Henry M. (2012). Emergence of the coherent structure of liquid water. Water.

[30] Yinnon C.A., Yinnon T.A. (2009). Domains in aqueous solutions: Theory and experimental evidence. Mod. Phys. Lett. B.

[31] Sen S., Gupta K.S., Coey J.M.D. (2015). Mesoscopic structure formation in condensed matter due to vacuum fluctuations. Phys. Rev. B.

[32] Manzalini A., Galeazzi B. (2019). Explaining homeopathy with quantum electrodynamics. Homeopathy.

[33] Messori C. (2019). Deep into the water: Exploring the hydro-electromagnetic and quantum-electrodynamic properties of interfacial water in living systems. Open Access Libr. J..

[34] Scirè A. (2020). A Mesoscopic model for the collective dynamics of water coherence domains. arXiv.

[35] Konovalov A.I., Ryzhkina I.S. (2014). Formation of nanoassociates as a key to understanding of physicochemical and biological properties of highly dilute aqueous solutions. Russ. Chem. Bull..

[36] Sedlák M., Rak D. (2013). Large-scale inhomogeneities in solutions of low molar mass compounds and mixtures of liquids: Supramolecular structures or nanobubbles?. J. Phys. Chem. B.

[37] Meessen A. (2018). Water memory due to chains of nano-pearls. J. Mod. Phys..

[38] Bellavite P., Marzotto M., Olioso D., Moratti E., Conforti A. (2014). High-dilution effects revisited. 1. physicochemical aspects. Homeopathy.

[39] Yinnon T., Kalia K., Kikar D. (2017). Very dilute aqueous solutions-structural and electromagnetic phenomena. Water.

[40] Jerman I., Ružič R., Krašovec R., Škarja M., Mogilnicki L. (2005). Electrical transfer of molecule information into water, its storage, and bioeffects on plants and bacteria. Electromagn. Biol. Med..

[41] Ruzic R., Jerman I., Skarja M., Leskovar R., Mogilnicki L. (2008). Electromagnetic transference of molecular information in garden cress germination. Int. J. High Dilution Res..

[42] Montagnier L., Aissa J., Del Giudice E.D., Lavallee C., Tedeschi A., Vitiello G. (2011). DNA waves and water. J. Phys. Conf. Ser..

[43] Yinnon T. (2020). Liquids Prepared by Serially Diluting and Vigorously Shaking of Aqueous Solutions: Unveiling Effects of the Solute on Their Properties. Water.

[44] Gudkov S.V., Penkov N.V., Baimler I.V., Lyakhov G.A., Pustovoy V.I., Simakin A.V., Sarimov R.M., Scherbakov I.A. (2020). Effect of mechanical shaking on the physicochemical properties of aqueous solutions. Int. J. Mol. Sci..

[45] Bandyopadhyay P., Bera D., Das K., Kumar B., Das S., Bhar D.S., Manchanda R., Khurana A., Nayak D., Basu R. (2017). Vigorous Shaking Enhances Voltage and Power Generation in Polar Liquids due to Domain Formation as Predicted by QED. Water J..

[46] Esposito F., Wolf U., Baumgartner S. (2021). NMR relaxation time investigation of highly diluted aqueous solutions of silica-lactose. J. Mol. Liq..

[47] Tang B.Q., Li T., Bai X., Zhao M., Wang B., Rein G., Yang Y., Gao P., Zhang X., Zhao Y. (2019). Rate limiting factors for DNA transduction induced by weak electromagnetic field. Electromagn. Biol. Med..

[48] Rad I., Jalali K. (2018). Electronic transmission of antibacterial property into water at extremely low frequency range: A preliminary study. J. ACM.

[49] Kernbach S. (2022). Electrochemical characterization of ionic dynamics resulting from spin conversion of water isomers. J. Electrochem. Soc..

[50] Kernbach S., Kernbach O. (2022). Environment-dependent fluctuations of potentiometric pH dynamics in geomagnetic field. Electromagn. Biol. Med..

[51] Del Giudice E.D., Spinetti P.R., Tedeschi A. (2010). Water dynamics at the root of metamorphosis in living organisms. Water.

[52] Liboff A.R. (1985). Geomagnetic cyclotron resonance in living things. J. Biol. Phys..

[53] Liboff A.R. Ion Cyclotron Resonance Interactions in Living Systems. SIBE, ATTI IV, PAVIA. **2013**, *19*, 1–14. https://emmind.net/openpapers_repos/Applied_Fields-Experimental/ELF_LF_Effects/ELF-EMF/2013_Ion_Cyclotron_Resonance_interactions_in_living_systems.pdf.

[54] Del Giudice E.D., Fleischmann M., Preparata G., Talpo G. (2002). On the “unreasonable” effects of ELF magnetic fields upon a system of ions. Bioelectromagnetics.

[55] Zhadin M., Giuliani L. (2006). Some problems in modern bioelectromagnetics. Electromagn. Biol. Med..

[56] Sedlák M. (2006). Large-scale supramolecular structure in solutions of low molar mass compounds and mixtures of liquids: I. light scattering characterization. J. Phys. Chem. B.

[57] Sedlák M. (2006). Large-scale supramolecular structure in solutions of low molar mass compounds and mixtures of liquids: II. kinetics of the formation and long-time stability. J. Phys. Chem. B.

[58] Sedlák M. (2006). Large-scale supramolecular structure in solutions of low molar mass compounds and mixtures of liquids. III. correlation with molecular properties and interactions. J. Phys. Chem. B.

[59] Bunkin N.F., Shkirin A.V., Penkov N.V., Chirikov S.N., Ignatiev P.S., Kozlov V.A. (2019). The physical nature of mesoscopic inhomogeneities in highly diluted aqueous suspensions of protein particles. Phys. Wave Phenom..

[60] Chikramane P.S., Suresh A.K., Bellare J.R., Kane S.G. (2010). Extreme homeopathic dilutions retain starting materials: A nanoparticulate perspective. Homeopathy.

[61] Del Giudice E., Tedeschi A., Vitiello G., Voeikov V. (2013). Coherent Structures in Liquid Water Close to Hydrophilic Surfaces. J. Phys. Conf. Ser..

[62] Pollack G.H. (2013). The Fourth Phase of Water: Beyond Solid, Liquid, and Vapor.

[63] Germano R., Del Giudice E., de Ninno A., Elia V., Hison C., Napoli E., Tontodonato V., Tuccinardi F.P., Vitiello G. (2013). Oxhydroelectric effect in bi-distilled water. Key Eng. Mater..

[64] Yinnon T. (2018). Aqueous solutions and other polar liquids perturbed by serial dilutions and vigorous shaking: Analyses of their UV spectra. Water.

[65] Chai B., Zheng J., Zhao Q., Pollack G.H. (2008). Spectroscopic studies of solutes in aqueous solution. J. Phys. Chem. A.

[66] Hu Y., Zhang Y., Cheng Y. (2022). Kinetic insight on the long-range exclusion of dissolved substances by interfacial interactions of water and hydrophilic surface. J. Mol. Liq..

[67] Elia V., Oliva R., Napoli E., Germano R., Pinto G., Lista L., Niccoli M., Toso D., Vitiello G., Trifuoggi M. (2018). Experimental study of physicochemical changes in water by iterative contact with hydrophilic polymers: A comparison between cellulose and nafion. J. Mol. Liq..

[68] Ryzhkina I.S., Murtazina L.I., Kiseleva Y.V., Konovalov A.I. (2015). Self-Organization and Physicochemical Properties of Aqueous Solutions of the Antibodies to Interferon Gamma at Ultrahigh Dilution. Dokl. Phys. Chem..

[69] Ayrapetyan S.N., Amyan A.M., Ayrapetyan G.S., Pollack G.H., Cameron I.L., Wheatley D.N. (2006). The effects of static magnetic fields, low frequency electromagnetic fields and mechanical vibration on some physicochemical properties of water. Water and the Cell.

[70] Radin D., Lund N., Emoto M., Kizu T. (2008). Effects of distant intention on water crystal formation: A triple-blind replication. J. Sci. Explor..

