# Supplementary material for: Interactions of Highly Diluted Arnica montana Extract with Water Across Glass Interfaces
Source: Int J Mol Sci. 2025 Jan 27;26(3):1115. doi: 10.3390/ijms26031115 (PMC11817096; doi:10.3390/ijms26031115)
Supplement: Supplementary file 1 [file ijms-26-01115-s001.zip › Supplementary Materials/Table S2.pdf]

Table S2. This table qualitatively illustrates a comparison between the hypotheses and the observed outcomes for conductivity measurements across three pH receiver solutions for three different dilutions: D6, C30, and C200. The hypotheses, represented by blue arrows, indicate the assumed direction. The color coding reflects the observed effect relative to the hypothesis, with red indicating the opposite direction, green indicating agreement, and slashes denoting no specific outcome.

	**Conductivity**								
		**Acidic**			**Neutral**			**Alkaline**	
	**D6**	**C30**	**C200**	**D6**	**C30**	**C200**	**D6**	**C30**	**C200**
**Hypotheses**	↑	↑	↑	↑	↑	↑	↑	↑	↑
**Outcome**	↓	/	/	↓	↓	↑	↓	/	↓
