# Supplementary figures and images for: Interactions of Highly Diluted Arnica montana Extract with Water Across Glass Interfaces
Source: Int J Mol Sci. 2025 Jan 27;26(3):1115. doi: 10.3390/ijms26031115 (PMC11817096; doi:10.3390/ijms26031115)

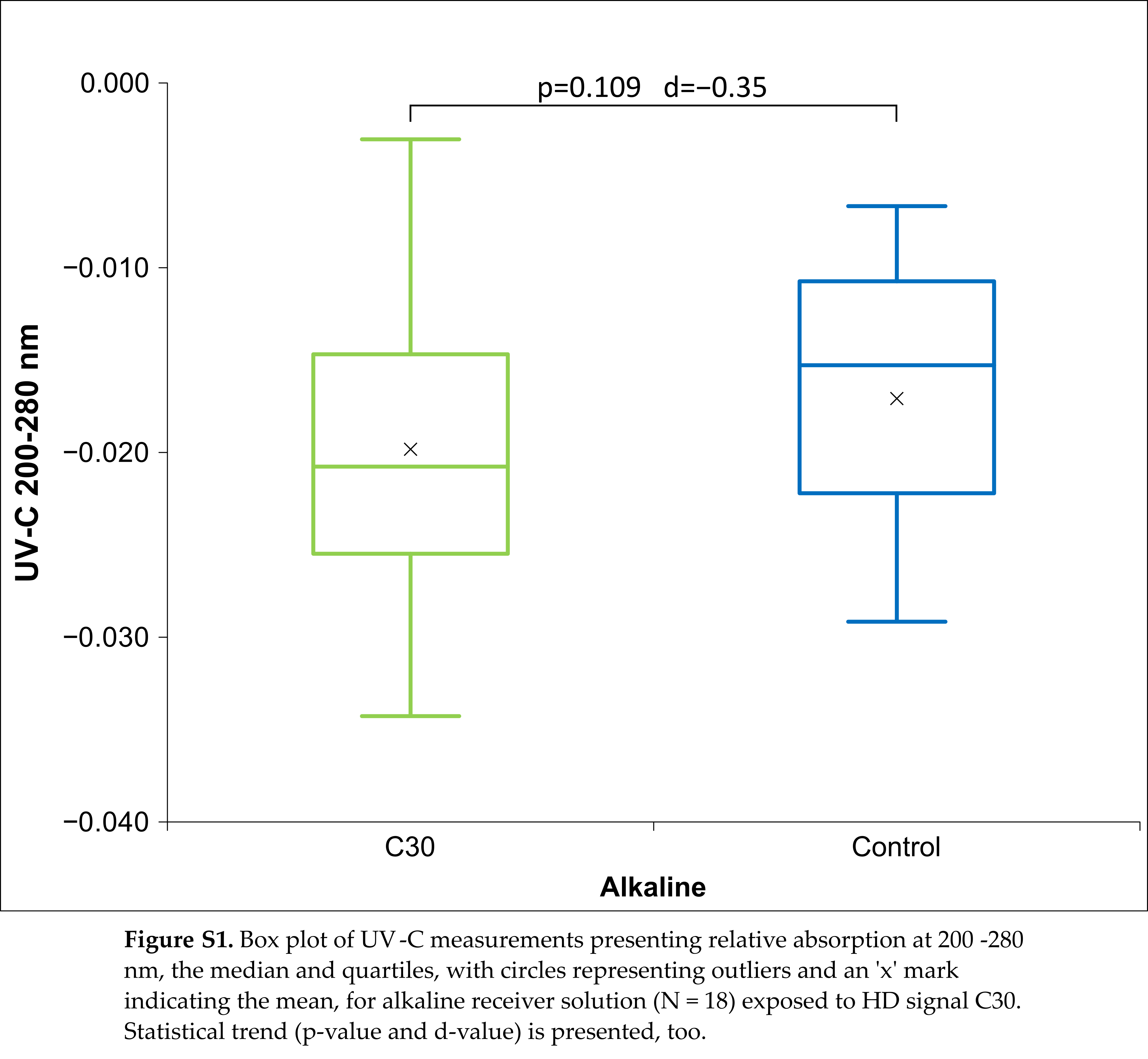

Supplement: Supplementary file 1 [file ijms-26-01115-s001.zip › Supplementary Materials/Supplementary Figure S1.png]

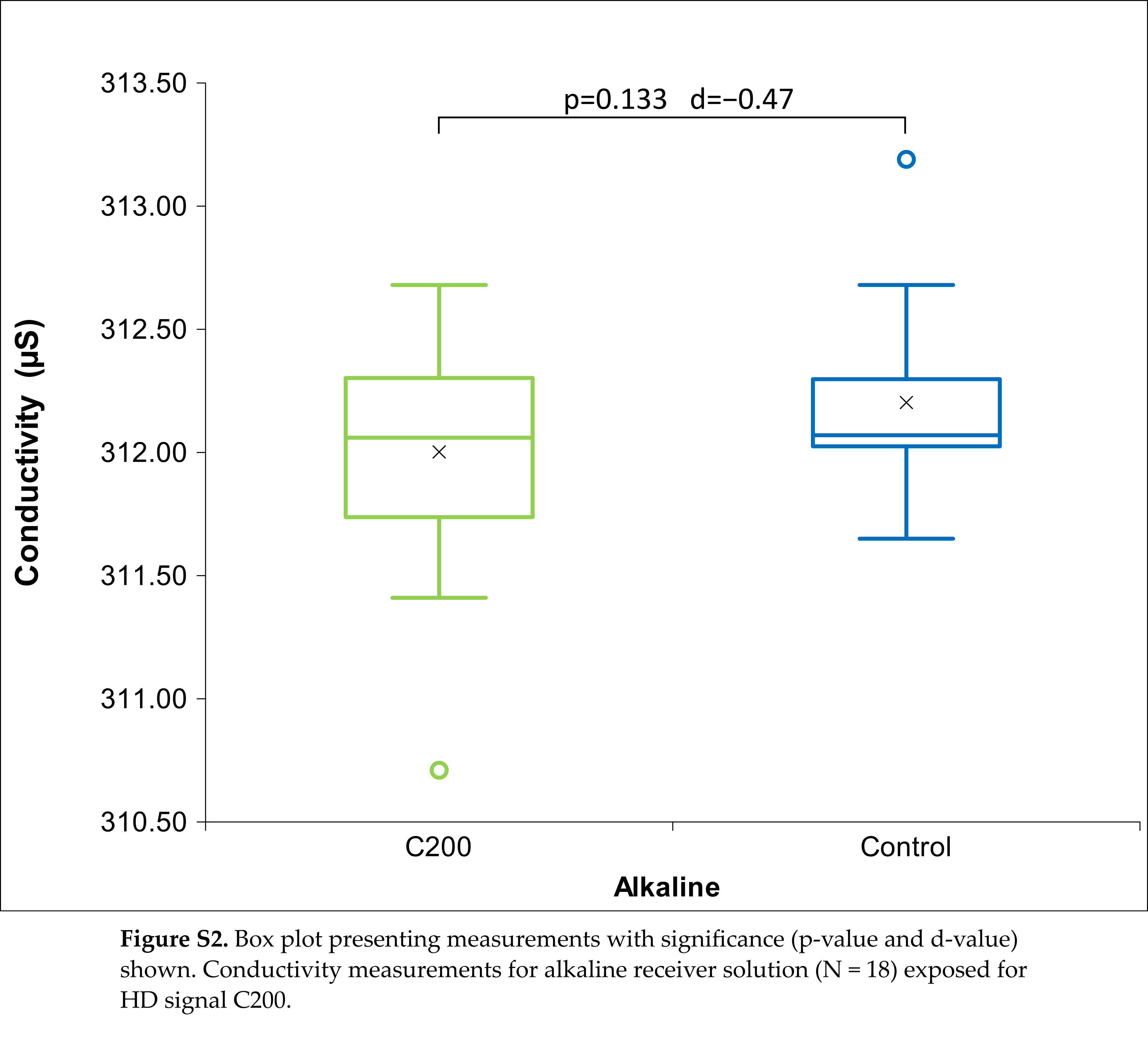

Supplement: Supplementary file 1 [file ijms-26-01115-s001.zip › Supplementary Materials/Supplementary Figure S2.png]
